# Mild manifestation of methanol poisoning half a day after massive ingestion of a fuel alcohol product containing 70% ethanol and 30% methanol: a case report

**DOI:** 10.1002/ams2.339

**Published:** 2018-04-10

**Authors:** Tomohiro Yoshizawa, Yoshito Kamijo, Yuji Fujita, Yoshiki Suzuki, Tomoki Hanazawa, Kiyotaka Usui, Tohru Kishino

**Affiliations:** ^1^ Department of Pharmacy Saitama Medical University Hospital Iruma‐gun Saitama Japan; ^2^ Emergency Medical Center & Poison Center Saitama Medical University Hospital Iruma‐gun Saitama Japan; ^3^ Department of Emergency, Disaster and General Medicine Iwate Medical University School of Medicine Morioka city Iwate Japan; ^4^ Department of Forensic Medicine Tohoku University Graduate School of Medicine Aoba‐ku Sendai Japan

**Keywords:** methanol poisoning, ethanol, hemodialysis, fomepizole, headspace gas chromatography/mass spectrometry

## Abstract

**Case:**

Is fomepizole necessary after massive ingestion of a mixture of methanol and ethanol? We report the case of a 37‐year‐old man who was transported to our Poison Center 12 h after ingesting 500 mL of fuel alcohol containing 70% methanol and 30% ethanol in a suicide attempt. On admission, he presented only with somnolence and mild metabolic acidosis. We hypothesized that most of the ethanol had been metabolized.

**Outcome:**

As the estimated serum concentration of methanol was lethal (242.6 mg/dL), fomepizole was given i.v. and hemodialysis was carried out twice, resulting in complete recovery. Later, the serum concentrations of both methanol and ethanol on admission were found to be 224.1 and 0.51 mg/dL, respectively.

**Conclusion:**

Therapeutic intervention was delayed by half a day after ingestion of a product containing methanol and ethanol in the present case. If the patient had arrived earlier, he may have only been treated with hemodialysis, but not fomepizole.

## Introduction

Fuel alcohol products containing methanol (MeOH) are commercially available as daily necessities in Japan. Since 2003, commercial fuel alcohol products that contain high concentrations of MeOH and ≥3% of ethanol (EtOH) have been regulated under the Act on the Quality Control of Gasoline and Other Fuels. However, the ratios of these components differ by manufacturer. Here we report a case in which a patient ingested a massive amount of a fuel alcohol product in a suicide attempt and was transported 12 h later to a poison center without serious signs or symptoms.

## Case

A 37‐year‐old man (height, 170 cm; weight, 96.7 kg) was transported to our Poison Center 12 h after ingesting 500 mL of a fuel alcohol product containing 70% MeOH and 30% EtOH in a suicide attempt. On arrival, his vital signs were: Glasgow Coma Scale, E3V4M6; heart rate, 88 b.p.m.; blood pressure, 158/117 mmHg; respiration rate, 15 breaths/min; SpO_2_, 98% (room air); and body temperature, 36.2°C. He had no remarkable medical history and did not take medication. Arterial blood gas findings were: pH 7.344; PaCO_2_, 31.7 mmHg; PaO_2_, 102 mmHg; ${\text{HCO}}_{3}^{-}$, 17.2 mmol/L; BE, −8.5 mmol/L. Plasma osmolality was 359 mOsm/kg, the osmolal gap (OG) was 75.8 mOsm/kg, and the anion gap was 13.8 mOsm/kg. Other blood laboratory findings were unremarkable.

We hypothesized that the patient had metabolized most of the EtOH because EtOH has a much higher affinity for alcohol dehydrogenase than MeOH and 12 h had already passed since ingestion of the product. The estimated serum concentration of MeOH (eMeOH) was calculated by 75.8 (OG) × 3.2 (molecular weight/10) to be 242.6 mg/dL. Given the diagnosis of lethal MeOH poisoning, repeated doses of fomepizole, an alcohol dehydrogenase inhibitor, were given i.v. and hemodialysis (HD) was intermittently carried out twice for 4 h each. After the first round of HD, OG and eMeOH decreased to 23.7 mOsm/kg and 75.8 mg/dL, respectively. During the second round of HD, OG and eMeOH decreased from 11.1 mOsm/kg and 35.5 mg/dL to 1.9 mOsm/kg and 6.0 mg/dL, respectively. As the patient was coherent and did not develop any serious signs or symptoms due to toxic metabolites of MeOH (e.g., vision abnormality), he was discharged on hospital day 4.

### Toxicological analysis

Serum samples were stored at −80°C. Serum concentrations of MeOH and EtOH were measured by headspace gas chromatography/mass spectrometry (HS‐GC/MS) (GCMS‐QP2020; Shimadzu, Kyoto, Japan). Sample injections into GC/MS were carried out using an HS sampler (HS‐20; Shimadzu) at 50°C for 60 min. The GC/MS analysis conditions were: column oven temperature, 50°C; and column flow rate, 2.43 mL/min. AQUATIC‐2 (0.25 mm I.D. × 60 m, df = 1.4 μm) (GL Sciences, Tokyo, Japan) was used for the separation column. Standard solutions containing 50–5,000 μg/mL of MeOH, 0.1–10 μg/mL of EtOH, and acetonitrile as an internal standard substance, were prepared, and a calibration curve was drawn by the internal standard method. Formic acid analysis was not possible due to the limited amount of sample. The MeOH, EtOH, and acetonitrile used for the analysis were purchased from Wako Pure Chemical Industries (Osaka, Japan).

## Results

On admission, MeOH and EtOH were 224.1 and 0.51 mg/dL, respectively. After the first round of HD, MeOH decreased to 93.9 mg/dL. After the second round of HD, MeOH decreased from 70.2 to 37.4 mg/dL.

## Discussion

Typical signs and symptoms of acute MeOH poisoning include metabolic acidosis, central nervous disorder, and vision abnormality caused by formic acid, a toxic metabolite of MeOH.^1^, ^2^ Rapidly absorbed from the gastrointestinal tract, MeOH is metabolized first to formaldehyde by alcohol dehydrogenase, and finally to formic acid by aldehyde dehydrogenase in the liver.^3^, ^4^, ^5^ If ingested in massive quantities, fomepizole, an alcohol dehydrogenase inhibitor, should be given as soon as possible before liver metabolism progresses, and HD should be carried out to remove MeOH.^6^, ^7^, ^8^, ^9^ Plasma osmolality is determined mainly by Na^+^, its counter ions, and uncharged species such as glucose and blood urea nitrogen. Plasma concentrations of these species allow for the accurate calculation of plasma osmolality. The difference between measured osmolality and calculated osmolality is referred to as OG. Alcohols can increase OG, and the estimated concentration of alcohols can be calculated by OG × molecular weight/10. As emergency facilities typically cannot determine MeOH concentrations, estimated MeOH (eMeOH) (OG × 3.2) has been used to evaluate the severity of poisoning and to determine whether HD should be undertaken. However, if a product containing MeOH and EtOH, both of which contribute to OG, is ingested, it may not be possible to determine eMeOH from OG.

In the present case, the patient showed only with mild central nervous system inhibitory symptoms, probably caused by MeOH itself, but did not develop serious signs or symptoms caused by formic acid, even after 12 h had passed from massive ingestion of the fuel alcohol product. It could be possible that EtOH, the affinity of which to alcohol dehydrogenase is much higher than that of MeOH, was metabolized first.^10^ Indeed, toxicological analysis revealed that EtOH was almost completely metabolized, but a lethal amount of MeOH remained in the patient's body. Although the serum concentration of formic acid was not determined due to the lack of residual sample, mild metabolic acidosis without AG suggested that the amount of formic acid was not large enough to cause serious signs or symptoms.

Therefore, for cases of delayed arrival to a poison center after massive ingestion of a product containing both MeOH and EtOH, it might be possible to evaluate the severity of MeOH poisoning because MeOH mostly contributes to OG. However, if EtOH remains in the body, eMeOH could be higher than MeOH. After HD sessions, MeOH is higher than eMeOH. The OG might have been affected by small uncharged species other than glucose and blood urea nitrogen, which can be easily eliminated by HD.

Thus, over‐triage may be a safer strategy for treatment. The time until manifestation of signs and symptoms caused by formic acid differs by individual and the ratio of MeOH and EtOH of the ingested product. Further studies on these aspects are warranted.

## Conclusion

The present case involved delayed treatment with fomepizole and HD by at least half a day after ingestion of a product containing 70% MeOH and 30% EtOH. The EtOH in the product may have effectively delayed MeOH metabolism. If the patient had arrived earlier after ingestion of the product, he might have been possibly treated only with HD, but not fomepizole.

## Disclosure

Approval of the research protocol: N/A.

Informed consent: Informed consent for publication of this case report was obtained from the subject.

Registry and the registration no. of the study/trial: N/A.

Animal studies: N/A.

Conflict of interest: None.
