# Corrigendum

**DOI:** 10.1002/ams2.742

**Published:** 2022-03-16

In T. Yoshizawa et al.,^1^ The authors would like to draw the reader’s attention for the error occurred in the title of published article:

‘Mild manifestation of methanol poisoning half a day after massive ingestion of a fuel alcohol product containing 70% ethanol and 30% methanol: a case report’ should be corrected to: ‘Mild manifestation of methanol poisoning half a day after massive ingestion of a fuel alcohol product containing 30% ethanol and 70% methanol: a case report’.

The authors apologise for this error and any confusion caused.
